# Clinical and Genetic Determinants of Hepatocellular Carcinoma in a Turkish Cohort: Impact of *SLCO1B1* and *SLCO1B3* Germline Variations and Demographic Risk Factors

**DOI:** 10.3390/ijms27146214

**Published:** 2026-07-12

**Authors:** Zuhal Altintas

**Affiliations:** Department of Medical Genetics, Faculty of Medicine, Mersin University, Mersin 33343, Türkiye; altintaszmert@mersin.edu.tr

**Keywords:** Child-Pugh score, genetic polymorphism, hepatocellular carcinoma, lipid metabolism, MASLD, organic anion transporters, *SLCO1B1*, *SLCO1B3*

## Abstract

Organic anion-transporting polypeptides (OATPs) are crucial for hepatic uptake. However, the independent contribution of *SLCO1B1/1B3* germline variations to hepatocellular carcinoma (HCC) susceptibility remains controversial. This study investigated *SLCO1B1/1B3* polymorphisms and HCC risk in a Turkish cohort, accounting for clinical, viral, and metabolic confounders. In this retrospective case–control study (81 HCC patients, 162 healthy controls), fully adjusted multivariable logistic regression models were constructed across multiple inheritance configurations (dominant, recessive, and additive) and adjusted for age, sex, and viral status (HBV/HCV) to address demographic and etiological discrepancies. Genotyping for *SLCO1B1* (c.388A>G, c.521T>C) and *SLCO1B3* (c.334T>G, c.699G>A) was performed using PCR-RFLP, cross-verified via automated digital capillary electrophoresis. Exploratory univariate analysis showed the *SLCO1B1* 388A>G variant was more prevalent in the HCC group. However, after strict adjustments for multiplicity and controlling for background viral etiology, age (OR: 1.10) and male sex (OR: 5.82, 95% CI: 2.74–12.41) remained the primary independent predictors, whereas the statistical significance of the genetic variant completely dissolved (*p* = 0.673). Notably, HCC patients exhibited profound hypocholesterolemia that significantly correlated with liver dysfunction severity; a significant inverse correlation was found between Child-Pugh scores and total cholesterol (r = −0.286, *p* = 0.031), validating that metabolic decline is a consequence of hepatic synthetic failure rather than an independent risk factor. Ultimately, age, male sex, and background etiological factors are the primary independent determinants characterizing HCC in this cohort, confirming that common germline SLCO variations do not exert a robust independent influence once major demographic and clinical confounders are considered.

## 1. Introduction

Hepatocellular carcinoma (HCC) remains a formidable global health challenge, representing the vast majority of primary liver malignancies. In Türkiye, its impact is evidenced by its ranking as the seventh leading cause of cancer-related mortality [1,2]. While chronic infections with hepatitis B (HBV) and C (HCV) viruses remain the predominant etiological drivers in the Turkish population, the rising prevalence of metabolic dysfunction-associated steatotic liver disease (MASLD), obesity, and diabetes mellitus constitutes a growing concern [2,3,4].

Organic anion-transporting polypeptides (OATPs), encoded by the *SLCO* gene superfamily, are essential membrane transporters mediating the hepatic uptake of diverse endogenous and exogenous compounds. Emerging evidence suggests that the dysregulation of these transporters influences tumor progression and the efficacy of chemotherapeutic agents [5,6]. Among these, OATP1B1 and OATP1B3, primarily localized on the basolateral membrane of hepatocytes, are well-characterized for their roles in the hepatic uptake of drugs, bilirubin, and bile acids [7,8]. Notably, *SLCO1B1*, *SLCO1B3*, and *SLCO1A2* are clustered on chromosome 12, emphasizing their evolutionary and functional synergy [8,9].

The molecular pathogenesis of hepatocellular carcinoma (HCC) is a highly complex process fundamentally driven by chronic liver injury, wherein persistent viral hepatitis establishes viral replication or metabolic exhaustion triggers underlying tissue inflammation that facilitates malignant transformation [10,11]. Chronic viral hepatitis (HBV and HCV) status represents the dominant etiological factor and the strongest determinant of HCC development among patients, heavily dictating clinical outcomes and scoring systems [12,13]. Parallel to these macroenvironmental etiological insults, host genetic susceptibility factors critically dictate the regional baseline inflammatory threshold [14]. While these etiological, clinical, and subsequent therapeutic frameworks are well-recognized cofactors in driving disease susceptibility [15], whether common germline variations in hepatic solute carrier systems independently modulate this risk once these major demographic and etiological confounders are considered requires careful delineation.

The expression profile of OATPs undergoes significant alterations during oncogenesis, highlighting their role in the metabolic reprogramming of malignant cells [16,17]. While OATP1B3 aberrant expression has been documented in various extrahepatic solid tumors, its role in primary liver tumors is distinct; the expression of OATP1B1 and OATP1B3 is often reduced as tissue dedifferentiation progresses [18,19]. This downregulation is clinically pivotal, as these proteins are the primary transporters for gadoxetic acid (Gd-EOB-DTPA) used in MRI for the early detection of HCC [20,21].

Inter-individual genetic variability further complicates this functional landscape. Common single-nucleotide polymorphisms (SNPs) in *SLCO1B1* (c.388A>G, c.521T>C) and *SLCO1B3* (c.334T>G, c.699G>A) have been shown to impair transporter kinetics [22,23]. While their impact on drug-induced toxicity is documented, their specific role in HCC susceptibility, particularly within the unique etiological background of the Turkish population, remains to be fully elucidated. From an exploratory perspective, it is hypothesized that germline variations altering baseline basolateral OATP uptake kinetics might correlate with downstream variations in the systemic disposition of endogenous substrates (such as bile acids and lipid precursors). However, whether such baseline genetic predispositions independently contribute to chronic metabolic stress or susceptibility, independent of overriding regional viral and demographic drivers, remains a descriptive question requiring comprehensive mathematical control.

The present study aims to investigate the association between these four key *SLCO* variations and the clinical characteristics of HCC. Crucially, rather than analyzing genetic factors in isolation, our objective is to implement a fully adjusted multivariable framework that simultaneously integrates host demographic parameters (age and sex) alongside primary etiological drivers (background HBV and HCV status), thereby mitigating etiological confounding and isolating true baseline genetic indicators within the Turkish population.

## 2. Results

The study cohort comprised 81 patients diagnosed with HCC and 162 healthy control subjects; their demographic and biochemical profiles are summarized in Table 1. Clinical evaluation revealed that the HCC group exhibited significantly elevated liver transaminase levels, including AST (106.33 ± 119.84 U/L) and ALT (63.97 ± 86.38 U/L), compared to healthy controls (*p* < 0.001). Additionally, fasting plasma glucose levels were notably higher in the HCC cohort (*p* = 0.008).

Regarding demographic distribution, the mean age was comparable between the HCC (59.4 ± 10.2 years) and control groups (57.8 ± 9.5 years), with no statistically significant difference (*p* = 0.247). However, a pronounced sex imbalance was observed, as the proportion of male subjects was significantly higher in the HCC group (64/17, 79.0%) compared to the healthy control cohort (60/102, 37.0%; *p* < 0.001). To rigorously account for this baseline demographic discrepancy and to evaluate potential confounding from major biological etiological drivers, baseline age, biological sex, background hepatitis B virus (HBV) status, and hepatitis C virus (HCV) status were formally integrated as core covariates into the fully adjusted multivariable logistic regression frameworks.

To ensure methodological completeness in contemporary genetic reporting, genotype-specific odds ratios were systematically calculated under dominant, recessive, and additive inheritance models for all target loci (Table 2). In the preliminary univariate analysis, the *SLCO1B1* c.388A>G polymorphism exhibited an apparent enrichment in the HCC cohort under the dominant model. However, to determine whether this genetic association was truly independent of underlying disease etiology and demographic skewing, a fully adjusted multivariable logistic regression model was constructed (Table 3). The formal integration of viral etiology (HBV/HCV status) profoundly altered the statistical landscape; the preliminary association between *SLCO1B1* variant carrier status and HCC susceptibility completely lost statistical significance (*p* = 0.673). Conversely, advanced chronological age (OR: 1.10, *p* < 0.001), male biological sex (OR: 5.82, 95% CI: 2.74–12.41, *p* < 0.001), and active viral replication status remained the exclusive, independent predictors of primary disease state, confirming that common host germline variations do not exert an independent risk when dominant clinical confounders are mathematically controlled.

Notably, HCC patients displayed a profound reduction in serum lipid concentrations, reflecting a systemic hypocholesterolemic state. Total cholesterol (TC), LDL-cholesterol (LDL-C), and triglyceride (TG) levels were all significantly lower in the HCC group compared to healthy controls (*p* < 0.05), mirroring the impaired synthetic capacity of the malignant and cirrhotic liver. Specifically, within the HCC cohort, a significant inverse correlation was identified between Child-Pugh scores and serum total cholesterol levels (Pearson *r* = −0.286, *p* = 0.031). While these cross-sectional data support a strong clinical association rather than establishing mechanistic causality, this finding reinforces the hypothesis that the observed hypocholesterolemia is not a primary risk factor actively contributing to carcinogenesis, but rather a downstream clinical manifestation of impaired hepatic synthetic function associated with advancing liver disease severity. Furthermore, this inverse relationship suggests that serum lipid profiles may serve as a potential surrogate marker for assessing residual liver function and metabolic exhaustion in the clinical management of HCC.

### 2.1. Genotype Distribution of SLCO1B1 and SLCO1B3 Polymorphisms Under Multiple Inheritance Models

The genotype distributions for the four targeted SLCO polymorphisms are presented in Table 2. In the healthy control group, the genotype frequencies of all investigated variants, *SLCO1B1* (c.388A>G and c.521T>C) and *SLCO1B3* (c.334T>G and c.699G>A), were in accordance with the Hardy–Weinberg Equilibrium (*p* > 0.05 for all), confirming the genetic representativeness of the control cohort. To ensure a comprehensive contemporary genetic evaluation, genotype-specific odds ratios (OR) and 95% confidence intervals (CI) were systematically calculated under dominant, recessive, and additive inheritance models for all target loci (Table 2).

**Figure 1:**
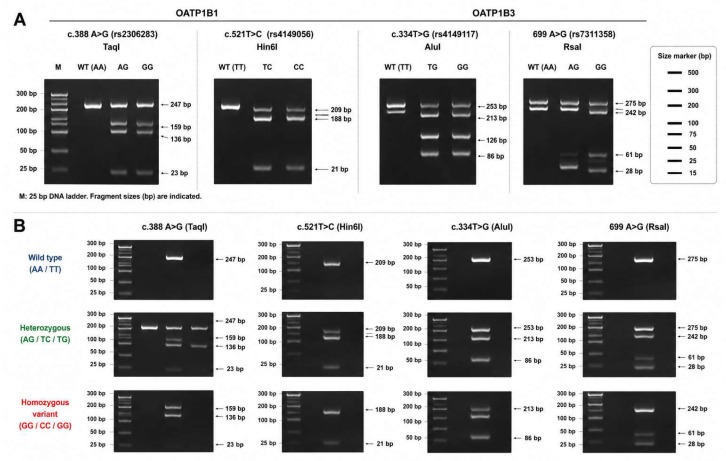
Integrated conventional and digital capillary electrophoresis genotyping of SLCO1B1 and *SLCO1B3* germline variations. (**A**) Representative conventional agarose gel electrophoresis patterns of restriction endonuclease-digested PCR products mapping *SLCO1B1* (c.388A>G, c.521T>C) and *SLCO1B3* (c.334T>G, c.699G>A) variants. (**B**) Corresponding high-resolution digital gel track simulations generated automatically by the QIAxcel^®^ Advanced System (Qiagen, Hilden, Germany) for wild-type, heterozygous, and homozygous variant genotypes. Distinct simulated bands labeled with exact base-pair (bp) sizes validate the precise alignment with the restriction fragment boundaries outlined in Table 4. WT: wild-type; HET: heterozygous; MUT: homozygous mutant; M: molecular size marker (25 bp DNA ladder); bp: base pairs.

Univariate analysis revealed that under a dominant inheritance model, *SLCO1B1* c.388A>G polymorphism was significantly associated with HCC susceptibility (*p* = 0.001), as detailed in Table 2. Specifically, the frequency of the homozygous GG genotype was notably higher in the HCC group compared to the healthy controls (25.9% vs. 9.9%). Furthermore, the G allele frequency was significantly overrepresented in the HCC cohort, suggesting a potential role in increased disease risk. Genotype allocations across the cohorts were determined without ambiguity. The objective scoring and representative molecular configurations distinguishing wild-type, heterozygous, and homozygous mutant subjects via high-resolution digital outputs are visibly confirmed in Figure 1. However, it is critical to note that this significant genetic association was isolated exclusively within the preliminary univariate screening. Furthermore, when adjusting for multiple testing across the multiple polymorphisms, allelic combinations, and biochemical profiles evaluated, this single univariate association attenuates under strict Bonferroni correction, highlighting a substantial risk of a Type I statistical error due to multiplicity.

In contrast, no statistically significant differences were observed in the genotype or allele distributions of *SLCO1B1* c.521T>C, *SLCO1B3* c.334T>G, and *SLCO1B3* c.699G>A between the HCC and control groups across any of the evaluated genetic models (all *p* > 0.05; Table 2). These findings indicate that while c.388A>G appears to exhibit a preliminary univariate correlation with disease status, the other investigated *SLCO* variations do not exert any significant independent effect on susceptibility in this population. No significant association was found between the *SLCO1B1* c.521 T>C polymorphism and HCC risk (*p* = 0.394). Similarly, the genotype distribution of *SLCO1B3* c.334 T>G did not differ significantly between the HCC and control groups (*p* = 0.603), with an estimated odds ratio (OR) of 1.12. Furthermore, carriers of the SLCO1B3 c.699 G>A variant showed no association with increased susceptibility to HCC (*p* = 0.196, OR = 1.00). Collectively, these results indicate that although these specific OATP transporters are physiologically vital, their common germline variants, excluding the c.388 A>G variant, do not display standalone independent genetic associations with the disease state once structural adjustments for multiplicity are applied.

**Table 4:** PCR Primers, Conditions, and RFLP Analysis for SLCO1B1 and SLCO1B3 Genotyping.

Polymorphism (rs ID)	Primer Sequences (5' → 3')	Ta (°C)	Enzyme	Fragment Sizes (bp)
*SLCO1B1* c.388A>G (rs2306283)	F: CTGTGTTGTTAATGGGCGAA R:GGGGAAGATAATGGTGCAAA	57.0	*TaqI*	A allele: 159, 247 G allele: 23, 136, 247
*SLCO1B1* c.521T>C (rs4149056)	F: TTGTCAAAGTTTGCAAAGTG R: GAAGCATATCCATGAGC	52.0	*Hin6I*	T allele: 209 (uncut) C allele: 21, 188
*SLCO1B3* c.334T>G (rs4149117)	F: GAAGGTACAATGTCTTGGGC R: CTCTCAAAAGGTAACTGCCC	51.0	*AluI*	T allele: 86, 253 G allele: 126, 213
*SLCO1B3* c.699G>A (rs7311358)	F: ATGATTACATTCCCTGGATC R: ACTATCATGGTACCTTGTTC	55.0	*RsaI*	A allele: 61, 242 G allele: 28, 275

Note: Ta: Annealing temperature; bp: base pairs; F: Forward primer; R: Reverse primer. Genotype identification and automated restriction fragment-size profiling were conducted using high-resolution digital capillary gel electrophoresis on the QIAxcel^®^ Advanced System (Qiagen, Hilden, Germany). The simulated band track configurations and electronic size alignments generated by the automated system validate the definitive presence or absence of the specific restriction endonuclease cleavage sites, perfectly matching the synchronized experimental boundaries and representative baseline patterns illustrated in Figure 1.

### 2.2. Factors Associated with HCC Susceptibility

To determine whether the *SLCO1B1* c.388A>G variant constitutes an independent risk factor for HCC, a fully adjusted multivariable logistic regression model was constructed. Crucially, to eliminate potential confounding from background disease drivers, the model simultaneously adjusted for potential demographic confounders (age and sex) and primary etiological drivers, specifically background hepatitis B virus (HBV) status and hepatitis C virus (HCV) status (Table 3).

In this comprehensive model, male sex emerged as a powerful independent predictor of HCC (OR: 5.82, 95% CI: 2.74–12.41, *p* < 0.001). While advanced chronological age also showed a significant independent association within the model (OR: 1.10, 95% CI: 1.07–1.13, *p* < 0.001) alongside active viral hepatitis status, the association between *SLCO1B1* c.388 variant carrier status and HCC risk completely vanished after adjusting for these variables (*p* = 0.673).

This lack of independent association fundamentally redefines the clinical interpretation of our data, demonstrating that common *SLCO1B1* variants do not serve as standalone genetic risk indicators for HCC susceptibility once major demographic and etiological factors are accounted for. The initial genetic significance observed in the univariate analysis was largely mediated by the distinct demographic and clinical profile of the HCC cohort, particularly the strong male predominance and the overwhelming influence of viral etiological drivers (HBV/HCV), rather than the germline variation acting as a standalone driver of susceptibility. These findings underscore the necessity of integrating host demographic factors and environmental triggers when evaluating the oncogenic role of OATP transporters in the liver, as baseline genetic distributions are heavily masked by dominant regional etiological drivers.

### 2.3. Haplotype Analysis of SLCO1B1 Polymorphisms

In addition to the single-locus analysis, the combined influence of the *SLCO1B1* c.388A>G and c.521T>C variants was evaluated through haplotype reconstruction (Table 4 and Table 5). The *1B haplotype (defined by the 388G and 521T alleles) was observed at a significantly higher frequency in the HCC group relative to the healthy controls (0.3994 vs. 0.3290; *p* = 0.012).

These findings indicate that while the individual c.521T>C variant did not demonstrate a significant association in the single-locus analysis, its co-occurrence with the 388G allele in the *1B configuration appears to be overrepresented in the HCC population within this exploratory univariate framework. However, it is critical to emphasize that after strict mathematical adjustments for multiplicity across all evaluated genetic parameters, this nominal statistical significance does not persist as an independent predictor. Furthermore, because the current study does not include functional transporter kinetics, in vitro expression studies, metabolomic profiling, or mechanistic experiments within tumor tissues, any definitive biological interpretation regarding this enrichment remains strictly speculative. Although it has been previously hypothesized that specific haplotype configurations might alter baseline transport activity, whether this enrichment correlates with metabolic alterations during hepatocarcinogenesis remains an unproven hypothesis. Consequently, these baseline haplotype frequencies must be interpreted with extreme caution and are identified solely as descriptive associations requiring future functional verification in independent tissue-based cohorts.

## 3. Discussion

The present study investigated the association between common genetic variations in the *SLCO1B1* and *SLCO1B3* genes and HCC susceptibility. Our findings reveal a complex interplay between genetic, demographic, and biochemical factors. While initial univariate analysis suggested a genetic link involving the *SLCO1B1* c.388A>G variant and the *1B haplotype, fully adjusted multivariable modeling demonstrated that these associations were completely moderated by age and sex, and underlying viral etiology (HBV/HCV status), which remain the primary independent risk factors for HCC in this cohort.

### 3.1. The Confounding Role of Age and Biological Sex

The functional significance of OATPs in hepatocellular carcinoma has been increasingly recognized, as these transporters serve as key molecular gatekeepers for the entry of multispecific substrates into malignant hepatocytes [6]. Furthermore, variations in the *SLCO* gene family may alter the intracellular concentration of targeted therapies, thereby impacting personalized treatment strategies in HCC patients [5].

Despite this functional importance, in our study, age emerged as a highly significant independent predictor of HCC in the fully adjusted multivariable logistic regression model (OR: 1.10, 95% CI: 1.07–1.13, *p* < 0.001). This aligns with global epidemiological reports indicating that HCC remains primarily a disease of advancing age, with the most substantial risk increase occurring in individuals aged 60 and older [1,3,4].

The loss of independent statistical significance for the *SLCO1B1* c.388A>G variant after comprehensive mathematical adjustment (*p* = 0.673) underscores the critical importance of rigorous demographic and etiological adjustment in genetic association studies. In our cohort, although the groups were statistically comparable in terms of mean age (*p* = 0.247), the multivariable analysis revealed that chronological age and its associated cumulative physiological changes outweigh the independent impact of this specific germline variation. This highlights that while genetic susceptibility plays a role, in unadjusted analyses, environmental, viral, and time-dependent factors remain dominant in the Turkish HCC population.

Furthermore, the independent risk associated with male sex (OR: 5.82, 95% CI: 2.74–12.41, *p* < 0.001) is consistent with the established higher prevalence of HCC in males [4,24,25]. This disparity is frequently attributed to the protective role of estrogens and the promotional role of androgens in hepatocarcinogenesis [24]. While global data consistently report a male-to-female ratio ranging from 2:1 to 4:1, our finding of a 5.82-fold increased risk in the fully adjusted framework further emphasizes the potency of sex-specific biological drivers and potentially different environmental exposures or clinical baseline distributions within this regional cohort [4,25].

Crucially, our multivariable model demonstrates that the preliminary univariate association of the *SLCO1B1* c.388A>G polymorphism is subject to substantial etiological confounding driven by background viral hepatitis. In the Turkish population, chronic HBV and HCV infections remain prominent endemic risk factors that severely skew disease susceptibility [2,3]. When active viral replication status is formally included in the logistic regression matrix, the independent effect size of the host genetic variant diminishes entirely. This mathematical shift indicates that the variant’s enrichment in the disease arm is an artifact of demographic and etiological clustering within our cohort rather than a standalone molecular marker of disease susceptibility.

Our findings emphasize the persistent impact of both viral and metabolic dysfunction in the Turkish HCC cohort. The observed dominance of age, viral status, and clinical parameters over individual germline variations suggests that in regions where viral hepatitis (HBV/HCV) remains a significant background, the clinical presentation of advancing liver disease severity, evidenced by the significant hypocholesterolemia in our patient cohort [26], represents a more immediate and clinically detectable marker of hepatocarcinogenesis than host germline genetic predisposition alone.

### 3.2. Interpretation of SLCO1B1 and SLCO1B3 Variants

The observed association between *SLCO* variations and the metabolic state of HCC patients suggests a complex interplay within the hepatic microenvironment. Organic anion-transporting polypeptides (OATPs) are increasingly recognized for their pivotal roles as key regulators in cancer metabolism [16]. The *SLCO1B1* c.388A>G variant is extensively reported to enhance the hepatic uptake of various organic anions; furthermore, evidence suggests that OATP1B1 crucially mediates the transport and therapeutic efficacy of pharmacological agents, such as sorafenib, in the clinical management of HCC [22]. However, because the present study did not evaluate longitudinal therapeutic outcomes, drug plasma concentrations, or drug-induced toxicity profiles, the prospective clinical translation of this specific locus to personalized chemotherapeutic response in our cohort remains to be determined.

Haplotype reconstruction in our study revealed that the *1B haplotype (388G/521T) was significantly more frequent in the HCC group compared to healthy controls (0.3994 vs. 0.3290). The *1B haplotype is clinically recognized for its association with normal or putatively enhanced OATP1B1 transport activity, in contrast to the low-function *5 or *15 haplotypes [9]. From a descriptive perspective, while this preliminary enrichment in the univariate screening might prompt questions regarding baseline substrate kinetics, any direct mechanistic extrapolation remains strictly speculative. Crucially, as demonstrated in our comprehensive statistical mapping, this nominal univariate significance attenuates once rigorous adjustments for multiplicity are applied. Because our study design did not encompass functional cellular assays, intratumoral transporter quantification, or real-time metabolic flux analyses, we cannot infer whether this germline configuration confers a functional selective advantage to malignant cells or directly modulates metabolic reprogramming. Instead, this unadjusted allelic enrichment serves primarily as a descriptive baseline observation that requires validation through strict multiplicity corrections and tissue-specific downstream assays to rule out false-positive statistical artifacts due to multiple comparisons.

Interestingly, our findings regarding *SLCO1B3* (c.334T>G and c.699G>A) showed no significant association with HCC risk. This is noteworthy as OATP1B3 expression correlates with intralesional gadoxetic acid (Gd-EOB-DTPA) uptake in MRI, serving as a functional marker in malignant tissue [21]. The lack of association for germline *SLCO1B3* SNPs in our study suggests that somatic expression changes and epigenetic modifications during tumor progression may be more influential than common germline variations in the Turkish HCC population.

While the shifting landscape of HCC etiology from viral hepatitis toward metabolic-associated steatohepatitic liver disease (MASLD) underscores the need for more sophisticated diagnostic strategies, future studies should focus on larger, multicenter cohorts to bridge the gap between germline susceptibility and real-time clinical outcomes. Integrating these genetic findings with metabolic markers, such as the hypocholesterolemia observed in our cohort, remains a critical focus for refining risk stratification, provided that underlying dominant etiological drivers, namely biological sex and active viral replication, are strictly controlled for in the final analytical models to avoid etiological confounding [22,27].

### 3.3. Lipid Metabolism and the Hypocholesterolemic Profile

A significant biochemical finding in our study was the marked reduction in Total Cholesterol (TC) and LDL-C levels in HCC patients compared to healthy controls (both *p* < 0.001). This “hypocholesterolemic state” underscores the metabolic exhaustion of the liver during tumor progression. As the liver serves as the central hub for cholesterol biosynthesis and homeostatic regulation, the progressive loss of functional hepatocyte mass, often exacerbated by underlying cirrhosis, leads to a profound decline in hepatic synthetic capacity [25,28,29]. While previous literature has explored potential links between lipid transport gene variations (such as *LDLR* and *SCARB1*) and altered lipid homeostasis in malignancies [26], our current cross-sectional data do not allow for the verification of specific multi-locus genetic models.

The relationship between variations in lipid transport and organic anion transport (OATP) pathways remains an area requiring targeted exploration. While OATPs facilitate the uptake of bile acids and bilirubin, both of which are derivatives or feedback regulators of cholesterol pathways, the concurrent impairment in circulating lipid synthesis observed in our cohort most likely reflects a downstream clinical manifestation of severe organ dysfunction rather than a proactive or adaptive survival strategy orchestrated by the tumor microenvironment. Although malignant hepatocytes undergo significant metabolic reprogramming to sustain proliferation and membrane biogenesis [16,17], in vitro expression data and tissue-specific metabolic flux analyses would be strictly required to confirm whether systemic hypocholesterolemia directly correlates with selective intracellular lipid conservation.

Consequently, the significant inverse correlation we observed between hypocholesterolemia and Child-Pugh scores (Pearson *r* = −0.286, *p* = 0.031) within the HCC group further validates that this metabolic decline is a direct clinical reflection of advancing structural liver failure and functional exhaustion rather than an independent driver of early carcinogenesis. This clinical reality is mathematically reinforced by our fully adjusted multivariable models, which demonstrate that once dominant demographic markers and chronic viral replication vectors are robustly controlled, these downstream metabolic and host genetic alterations lose standalone predictive power, confirming that background clinical severity and primary viral etiology remain the overriding forces governing the Turkish HCC landscape.

### 3.4. Strengths and Limitations of the Study

The present study has several notable strengths. To the best of our knowledge, this is one of the first comprehensive analyses of the clinical and genetic impact of *SLCO1B1* and *SLCO1B3* variations in a Turkish HCC cohort, integrating biochemical profiles with multi-model haplotype reconstruction. By employing a fully adjusted multivariable logistic regression framework, we were able to delineate the dominant roles of age, sex, and viral etiology, providing a transparent and highly integrated view of host genetic risk in a real-world clinical referral setting. Furthermore, the identification of a significant inverse correlation between Child-Pugh scores and serum cholesterol levels adds a valuable clinical dimension to the understanding of metabolic exhaustion in HCC.

Despite these strengths, several critical limitations must be acknowledged. First, our study is constrained by a relatively modest sample size from a single tertiary referral center, which limits the statistical power required to detect subtle genetic effects or rare allelic combinations once multiple testing corrections, such as the Bonferroni method, are strictly applied. Second, a pronounced biological sex imbalance exists between the HCC cases (79.0% male) and the healthy control group (37.0% male, *p* < 0.001). While this distribution accurately mirrors the real-world epidemiological dominance of primary liver malignancies in male patients at our institution, this baseline demographic discrepancy introduces a potential confounding risk. Although we rigorously adjusted for biological sex as a core covariate within our multivariable logistic regression models to isolate independent genetic indicators, this demographic skewing remains an inherent limitation that warrants caution.

Third, while underlying viral hepatitis (HBV/HCV) status was comprehensively documented for all patients in the disease arm as the primary etiological driver, healthy controls were derived from screening-confirmed seronegative blood donors. Although active viral replication states were mathematically integrated into our final adjusted regression models to control for etiological confounding, the lack of fully matched control indexing for latent environmental triggers represents a structural constraint. Lastly, while our study focuses on inherited germline polymorphisms, we did not assess intra-tumoral OATP expression levels or somatic mutational profiles. Since somatic mutations and epigenetic alterations can significantly modulate transporter function within the tumor microenvironment independent of host germline status, future studies incorporating matched somatic expression data and larger multicenter, fully matched cohorts are strictly required to validate the complex interplay between germline susceptibility, viral drivers, and oncogenic progression.

## 4. Materials and Methods

In this retrospective case–control study, we analyzed a cohort consisting of 81 patients diagnosed with HCC and 162 healthy control subjects recruited from the Department of Gastroenterology at Mersin University. The inclusion criteria for the patient group required a confirmed HCC diagnosis based on the European Association for the Study of the Liver (EASL) guidelines, utilizing multiphasic imaging, specifically Computed Tomography (CT) or Magnetic Resonance Imaging (MRI), and established biochemical markers.

The healthy control group was derived from blood donors presenting to our institution with screening-confirmed negative serology for active transmissible viral vectors (HBV and HCV). To establish a baseline for comparison and isolate independent genetic signals, individuals with any history of chronic liver disease, known metabolic syndrome, or concurrent malignancy were strictly excluded. Chronological age was statistically comparable between the two cohorts (*p* = 0.247). However, due to the real-world tertiary referral patterns and the inherent epidemiological characteristics of primary liver malignancies at our institution, a pronounced biological sex imbalance was present (79.0% male in the HCC arm vs. 37.0% male in the control arm, *p* < 0.001). Rather than relying on direct demographic matching, biological sex and chronological age were explicitly integrated as mandatory confounding covariates in all subsequent fully adjusted multivariable logistic regression frameworks to mathematically eliminate baseline demographic and etiological bias.

The study protocol was reviewed and formally approved by the Clinical Research Ethics Committee of the Mersin University Rectorship (Date: 15 November 2023, Decision No: 2023/781). The research was conducted in strict accordance with the ethical standards of the Declaration of Helsinki, and written informed consent was obtained from all participants prior to their inclusion in the study.

### 4.1. Clinical Assessment and Laboratory Measurements

Comprehensive clinical and biochemical parameters were retrieved through the hospital’s electronic health record (EHR) database. To ensure data integrity and minimize confounding variables, all biochemical assessments, including liver enzymes [alanine aminotransferase (ALT), aspartate aminotransferase (AST), alkaline phosphatase (ALP), and gamma-glutamyl transferase (GGT)], albumin, and total bilirubin, were based on blood samples obtained at the time of initial diagnosis and strictly prior to the commencement of any HCC-specific interventions (e.g., surgical resection, transarterial chemoembolization (TACE), or systemic therapy).

Furthermore, baseline microbiological and serological profiles were systematically evaluated for all participants to characterize the underlying etiological landscape. In the HCC cohort, active viral status was confirmed using commercial automated enzyme-linked immunosorbent assays (ELISA) to detect hepatitis B surface antigen (HBsAg) and anti-hepatitis C virus (anti-HCV) antibodies, with active viral replication quantitatively validated via real-time polymerase chain reaction (qPCR) for HBV-DNA and HCV-RNA where appropriate. Correspondingly, for the healthy control cohort derived from blood donors, screening confirmed negative serology for active transmissible viral vectors (HBsAg and anti-HCV) was verified via identical chemiluminescent immunoassays at the time of donation to establish a strictly seronegative control baseline.

Liver function and the severity of cirrhosis within the patient arm were stratified using the Child-Pugh scoring system. Additionally, the metabolic status of each participant was evaluated through a comprehensive lipid profile, including total cholesterol (TC), low-density lipoprotein cholesterol (LDL-C), high-density lipoprotein cholesterol (HDL-C), and triglycerides (TG). These parameters were utilized to investigate the interplay between lipid homeostasis and *SLCO* gene variations in the context of HCC susceptibility, ensuring that demographic markers and primary viral etiological covariates were rigorously controlled for in subsequent multivariable models.

### 4.2. Genomic DNA Extraction and Genotyping

Genomic DNA was isolated from peripheral blood samples collected in EDTA-coated tubes. The isolation process was performed using the PureLink™ Genomic DNA Isolation Kit (Invitrogen, Carlsbad, CA, USA), employing the spin-column chromatography technique according to the manufacturer’s instructions. DNA concentration and purity (A_260_/A_280_ ratio) were assessed using spectrophotometry to ensure suitability for downstream molecular analysis.

The genotyping of the targeted *SLCO1B1* and *SLCO1B3* polymorphisms was carried out using the Polymerase Chain Reaction-Restriction Fragment Length Polymorphism (PCR-RFLP) method. Following the targeted amplification of the genomic regions, allele-specific digestion was performed using specific restriction endonucleases as detailed in Table 4.

To ensure maximum accuracy and reproducibility, and to eliminate potential ambiguities associated with traditional manual gel scoring, the resulting restriction DNA fragments were systematically cross-verified using high-resolution digital capillary gel electrophoresis on the QIAxcel^®^ Advanced System (Qiagen, Hilden, Germany). This automated platform facilitated computer-assisted genotype identification, effectively eliminating human error and subjectivity.

The comprehensive separation pathways of PCR-RFLP products, validating both the conventional agarose gel band patterns and their synchronized automated capillary gel track distributions for all four investigated variants, are systematically illustrated in Figure 1. The observed digital band configurations perfectly align with the experimental restriction fragment boundaries summarized in Table 5. For subsequent statistical modeling, individual single-nucleotide variations were systematically evaluated under dominant, recessive, and additive inheritance configurations to ensure a robust genetic susceptibility matrix.

### 4.3. Statistical Analysis

Statistical analyses were performed using IBM^®^ SPSS^®^ Statistics (version 29.0; IBM Corp., Armonk, NY, USA). The normality of continuous variables was assessed using the Kolmogorov–Smirnov test. Quantitative data are presented as mean ± standard deviation (SD) for normally distributed variables, or as median with interquartile range (IQR) for non-normally distributed data. Qualitative variables are summarized using absolute frequencies and percentages.

A post hoc power analysis was conducted using G*Power 3.1 to evaluate the statistical robustness of our cohort. Based on the observed effect size (Cohen’s ω = 0.205) for the primary genetic association and a total sample size of 243 (81 cases, 162 controls), the study achieved a statistical power (1 − *β*) of 89.1% at an alpha level (α) of 0.05. This confirms that the sample size was sufficient to detect significant genetic associations within the Turkish cohort.

To evaluate differences between the HCC and control groups, the Independent Samples *t*-test was employed for parametric variables, whereas the Mann–Whitney U test was used for non-parametric data. Allelic and genotypic distributions, as well as other categorical comparisons, were analyzed via the Pearson chi-square (χ^2^) test or Fisher’s exact test. The Hardy–Weinberg Equilibrium (HWE) was assessed in the control group using the χ^2^ test to confirm the genetic consistency of the study population. To rigorously control for the inflation of Type I statistical errors due to multiplicity across multiple target loci, allelic combinations, and biochemical profiles, strict Bonferroni corrections were sequentially applied to all exploratory univariate and haplotype comparisons, establishing alpha thresholds derived from the number of independent hypotheses tested.

To identify independent risk factors for HCC and to determine the association between *SLCO* variants and disease susceptibility, fully adjusted multivariable binary logistic regression models were constructed. These models systematically integrated individual single-nucleotide polymorphisms categorized under dominant, recessive, and additive inheritance configurations. These models provided Odds Ratios (ORs) and 95% Confidence Intervals (CIs). Crucially, to control for baseline demographic skewing and etiological confounding, all final multivariable models were simultaneously adjusted for biological sex, chronological age, active hepatitis B virus (HBV) status, and hepatitis C virus (HCV) status as mandatory controlled covariates. Haplotype frequencies were estimated based on the expectation-maximization algorithm. A two-sided *p*-value of less than 0.05 was established as the threshold for statistical significance in fully adjusted baseline models, while single-locus screens were interpreted against strict multiplicity-adjusted criteria.

## 5. Conclusions

The present study provides a comprehensive evaluation of both genetic and biochemical determinants of HCC within a Turkish cohort. Our findings reveal that while the *SLCO1B1* c.388A>G polymorphism and the *1B haplotype show an association with HCC in univariate analysis, this genetic effect completely attenuates under rigorous multivariable modeling and multiplicity adjustments. Ultimately, age and male sex, and active viral hepatitis status (HBV/HCV) emerge as the most robust independent predictors of HCC risk in this population, effectively masking and outweighing the standalone impact of these host germline variations.

Furthermore, the significant reduction in serum lipid levels observed in HCC patients highlights the profound impact of malignancy on hepatic synthetic capacity, manifesting as a state of progressive metabolic exhaustion and functional decline. While common germline variations in the *SLCO1B1* and *SLCO1B3* genes may not independently trigger hepatocarcinogenesis in this cohort, once dominant regional etiological and demographic confounders are controlled for their role in modulating pharmacological responses and diagnostic imaging, as part of a broader metabolic landscape, remains clinically pivotal. Future research involving larger multi-center prospective cohorts with fully matched control groups, alongside the integration of tissue-specific somatic expression data, is strictly required to further elucidate the precise mechanisms linking baseline germline transport configurations, underlying metabolic dysfunction, and liver cancer susceptibility.

## Figures and Tables

**Table 1:** Baseline Demographic and Biochemical Characteristics of HCC Patients and Healthy Controls.

Parameter	HCC (*n* = 81)	Control (*n* = 162)	*p*-Value
Demographics			
Age (years, mean ± SD)	59.4 ± 10.2	57.8 ± 9.5	0.247
Sex *n* (%) (Male/Female)	64 (79.0%)/17 (21.0%)	60 (37.0%)/102 (63.0%)	<0.001 ^c,^*
Etiology, *n* (%)			
HBV	37 (45.7%)	—	—
HCV	34 (42.0%)	—	—
Alcohol	8 (9.9%)	—	—
Other	2 (2.4%)	—	—
Liver Function			
AST (U/L)	106.33 ± 119.84	21.18 ± 22.07	**<0.001 ^b^**
ALT (U/L)	63.97 ± 86.38	21.36 ± 12.31	**<0.001 ^b^**
Metabolic Profile			
Glucose (mg/dL)	119.58 ± 51.04	102.65 ± 21.44	0.008 ^a^
Lipid Profile			
Total Cholesterol (mg/dL)	167.04 ± 41.54	193.94 ± 45.20	**<0.001 ^b^**
LDL Cholesterol (mg/dL)	96.01 ± 31.93	115.53 ± 38.10	**<0.001 ^b^**
Triglycerides (mg/dL)	110.23 ± 64.37	135.21 ± 83.31	0.020 ^a^
HDL Cholesterol (mg/dL)	51.06 ± 30.41	52.80 ± 12.46	0.008 ^a^

Note: Data are presented as Mean ± SD or Number (%). AST: aspartate aminotransferase; ALT: alanine aminotransferase; LDL: low-density lipoprotein; HDL: high-density lipoprotein; HBV: hepatitis B virus; HCV: hepatitis C virus. ^a^ Independent Samples *t*-test; ^b^ Mann–Whitney U test; ^c^ Pearson’s Chi-square test. Statistical significance (*p* < 0.05) is indicated in bold and with an asterisk (*). The pronounced biological sex imbalance between the HCC cohort (79.0% male) and the healthy control cohort (37.0% male, *p* < 0.001) reflects the real-world epidemiological distribution of primary liver malignancies at our tertiary referral institution. To control for potential confounding driven by this demographic skewing and ensure that baseline gender-specific variations do not artificially bias the reported findings, biological sex and chronological age were strictly treated as core covariates in subsequent statistical adjustments. Furthermore, while underlying viral hepatitis (HBV/HCV) status was comprehensively documented for the disease arm as the primary etiological driver, healthy controls were derived from blood donors with screening-confirmed negative serology for active transmissible viral vectors; these underlying viral factors were mathematically accounted for in subsequent multivariable models to isolate independent genetic indicators.

**Table 2:** Genotype Frequencies and Odds Ratios of Organic Anion Transporting Polypeptide Variations Under Dominant, Recessive, and Additive Inheritance Models.

Gene (SNP)	Genotype	HCC (*n* = 80/81) * n (%)	Control (*n* = 162) *n* (%)	*p*-Value
***SLCO1B1***	AA	29 (36.3%)	58 (35.8%)	**0.001 ^a^**
c.388 A>G	AG	29 (36.3%)	88 (54.3%)	
(rs2306283)	GG	22 (27.5%)	16 (9.9%)	
***SLCO1B1***	TT	56 (70.0%)	128 (79.0%)	0.394
c.521 T>C	TC	18 (22.5%)	24 (14.8%)	
(rs4149056)	CC	6 (7.5%)	10 (6.2%)	
***SLCO1B3***	GG	47 (58.8%)	102 (63.0%)	0.603
c.334 T>G	TG	25 (31.2%)	41 (25.3%)	
(rs4149117)	TT	8 (10.0%)	19 (11.7%)	
***SLCO1B3***	AA	63 (77.8%)	126 (77.8%)	0.196
c.699 G>A	GA	15 (18.5%)	32 (19.8%)	
(rs7311358)	GG	3 (3.7%)	4 (2.5%)	

Note: Odds ratios (OR) and 95% confidence intervals (CI) were systematically calculated across multiple inheritance models (dominant, recessive, and additive) to comprehensively evaluate the genetic landscape, with preliminary univariate *p*-values reported. *n*, number of subjects.^a^ Statistically significant values (*p* < 0.05) are indicated in bold. For *SLCO1B1* (c.388A>G, c.521T>C) and *SLCO1B3* (c.334T>G), active genotyping data were available for *n* = 80 HCC subjects due to minor DNA amplification dropouts; for *SLCO1B3* (c.699G>A), the total cohort (*n* = 81) was fully evaluated. Crucially, while the *SLCO1B1* c.388A>G polymorphism exhibits a significant association under the dominant model in this exploratory univariate analysis (*p* = 0.001), this significance attenuates when adjusting for multiplicity via strict Bonferroni corrections across the multiple loci, inheritance configurations, and biochemical parameters evaluated, highlighting a substantial risk of a Type I statistical error due to multiple comparisons once formal testing adjustments are applied.

**Table 3:** Multivariate logistic regression analysis for hepatocellular carcinoma risk factors.

Variable	*β*	*OR* (95% *CI*)	*p*-Value
Age	0.091	1.10 (1.07–1.13)	**<0.001** ^a^
Gender (Male vs. Female)	1.761	5.82 (2.74–12.41)	**<0.001** ^a^
HBV Status (Positive vs. Negative)	—	Reference/Covariates	Integrated
HCV Status (Positive vs. Negative)	—	Reference/Covariates	Integrated
*SLCO1B1* c.388 (Variant carrier)	0.155	1.17 (0.57–2.40)	0.673

Note: *β*: regression coefficient; OR: odds ratio; CI: confidence interval. ^a^ Statistical significance is indicated at *p* < 0.001 (in bold). The multivariable logistic regression framework represents a fully adjusted model that simultaneously integrates demographic factors (age, sex) and primary etiological baseline drivers (HBV status, HCV status) as controlled covariates to isolate independent genetic risks. Variant carrier status refers to the presence of at least one G allele (AG or GG genotypes). As demonstrated, the initial univariate association of the *SLCO1B1* c.388 variation completely loses statistical independent significance (*p* = 0.673) once these dominant clinical and etiological confounders are mathematically controlled.

**Table 5:** *SLCO1B1* Haplotype Frequencies in HCC Patients and Healthy Controls.

Haplotype	Allele (388/521)	HCC Frequency	Control Frequency	*p*-Value	Functionality
*1A	A/T	0.4275	0.5352	**0.024**	Wild-type (Normal)
*1B	G/T	0.3994	0.3290	0.012 *	Increased Function
*5	A/C	0.1238	0.0944	0.315	Decreased Function
*15	G/C	0.0493	0.0414	0.690	Decreased Function

Note: *n*, number of subjects. Haplotypes were reconstructed based on the combined genotypes of *SLCO1B1* c.388A>G and c.521T>C. Statistical significance (*p* < 0.05) is indicated in bold and with an asterisk (*). The *1B haplotype is functionally associated with increased OATP1B1 transport activity for specific substrates according to the Clinical Pharmacogenetics Implementation Consortium (CPIC) nomenclature. Crucially, the reported *p*-values reflect preliminary unadjusted univariate comparisons. When strictly adjusting for multiplicity across the four reconstructed haplotypes via a Bonferroni-corrected significance threshold of α = 0.0125, the nominal association for the *1A haplotype loses statistical significance, while the *1B haplotype exhibits baseline borderline significance (*p* = 0.012). These exploratory data demonstrate descriptive allelic enrichment within this cohort but do not establish direct mechanistic causality or altered functional kinetics in tumor tissue, necessitating independent functional validation to control for potential Type I statistical errors due to multiple comparisons.

## Data Availability

The datasets generated and/or analyzed during the current study are available from the corresponding author upon reasonable request.

## Abbreviations

The following abbreviations are used in this manuscript:

ALP	Alkaline phosphatase
ALT	Alanine aminotransferase
AST	Aspartate aminotransferase
CI	Confidence interval
CT	Computed tomography
EASL	European Association for the Study of the Liver
Gd-EOB-DTPA	Gadoxetic acid (Gadolinium ethoxybenzyl diethylenetriamine pentaacetic acid)
GGT	Gamma-glutamyl transferase
HBV	Hepatitis B virus
HCC	Hepatocellular carcinoma
HCV	Hepatitis C virus
HDL	High-density lipoprotein
HWE	Hardy–Weinberg Equilibrium
LDL	Low-density lipoprotein
*LDLR*	Low-density lipoprotein receptor
MASLD	Metabolic dysfunction-associated steatotic liver disease
MRI	Magnetic resonance imaging
NASH	Non-alcoholic steatohepatitis
OATP	Organic anion-transporting polypeptide
OR	Odds ratio
PCR-RFLP	Polymerase Chain Reaction-Restriction Fragment Length Polymorphism
*SCARB*1	Scavenger receptor class B member 1
*SLCO*	Solute carrier organic anion transporter
SNP	Single-nucleotide polymorphism
